# Electrical Neuroimaging with Irrotational Sources

**DOI:** 10.1155/2015/801037

**Published:** 2015-05-31

**Authors:** Rolando Grave de Peralta Menendez, Sara Gonzalez Andino

**Affiliations:** ^1^Electrical Neuroimaging Group, 18 rue Albert Gos, 1206 Geneva, Switzerland; ^2^Neural Microcircuitry Lab, École Polytechnique Fédérale de Lausanne (EPFL), 1015 Lausanne, Switzerland

## Abstract

This paper discusses theoretical aspects of the modeling of the sources of the EEG (i.e., the bioelectromagnetic inverse problem or source localization problem). Using the Helmholtz decomposition (HD) of the current density vector (CDV) of the primary current into an irrotational (**I**) and a solenoidal (**S**) part we show that only the irrotational part can contribute to the EEG measurements. In particular we present for the first time the HD of a dipole and of a pure irrotational source. We show that, for both kinds of sources, **I** extends all over the space independently of whether the source is spatially concentrated (as the dipole) or not. However, the divergence remains confined to a region coinciding with the expected location of the sources, confirming that it is the divergence rather than the CDV that really defines the spatial extension of the generators, from where it follows that an irrotational source model (ELECTRA) is always physiologically meaningful as long as the divergence remains confined to the brain. Finally we show that the irrotational source model remains valid for the most general electrodynamics model of the EEG in inhomogeneous anisotropic dispersive media and thus far beyond the (quasi) static approximation.

## 1. Introduction

The irrotational source model was explicitly proposed as a biophysically constrained model for the EEG in [1, 2]. It simply reflects the physical fact that EEG (potential) measurements convey no information about solenoidal sources. On this basis, we stated the inverse bioelectromagnetic problem solely in terms of the irrotational part of the currents and further showed that the resulting inverse problem can be reduced to the determination of potentials within the brain (ELECTRA).

While the irrotational source model is a rather trivial consequence of basic textbook results, it can, nevertheless, lead to an apparent contradiction when we try to place it within the context of EEG models. The basic problem arises because we intuitively expect the generators (i.e., the neural currents) to be confined to the brain volume. Yet, irrotational currents are different from zero whenever the conductivity is different from zero. Consequently, the irrotational sources are not confined to the gray cortical matter but rather extend into tissues where no primary currents are expected as, for example, white matter, bone, or the cerebrospinal fluid. We will here show that this observation is not in contradiction with the physical and physiological validity of the irrotational source model (ISM) of ELECTRA. In fact, we will formally show that the spatial extent of the generators is defined by its divergence rather than by the spatial extent of its irrotational component. We will also show that the divergence of the irrotational component of focal (dipolar) sources remains confined to the dipole center while the divergence of the irrotational component of extended sources confines itself to the brain region. This observation helps to understand the measure we should minimize within inverse problems to restrict the spatial extent of biological sources.

To formally introduce the problem let us start with the following example fully justified by results in [3]. Let us consider a region *B* of the 3D space and a function *ρ*(**r**) with continuous first order partial derivatives inside *B* and continuous on the closure of *B* (i.e., the union of *B* and its frontier). Then, we can define the two functions *V*(**r**) and **J**(**r**) as follows:

(1) Vr−14π∫Bρpr−pdpJr=∇Vr.

Noting that the derivatives of *V* can be computed by differentiation under the integral sign with respect to variable **r**, it results that **J**(**r**) is, by construction, irrotational and continuous throughout the 3D space (*ℜ* ^3^) and with continuous partial derivatives inside *B*.

If we interpret region *B* as the brain in an infinite and homogeneous medium with constant conductivity *σ* (e.g., unitary) conductivity, then we have an irrotational current source density vector **J**(**r**) defined and derivable everywhere in the 3D space and generating potential *V*(**r**) which solves the mathematical problem of the EEG (i.e., Poisson equation with Dirichlet boundary conditions and *σ* = 1), as can be verified using identity (A.3) and the symmetry of the delta function (B.2) described in the Appendices A and B, respectively:

(2) $$\begin{array}{c} \nabla \cdot \left(\;\sigma \nabla V\left(\;r\;\right)\;\right)=\nabla^{2}V\left(\;r\;\right)=\nabla \cdot J\left(\;r\;\right)=\left\{\begin{array}{cc} \rho \left(r\right) & r\in B\\ 0 & r\notin B. \end{array}\right. \end{array}$$

Equations (1) provide a simple example of a current source density vector which is by construction irrotational since it is defined as the gradient of a voltage generated by a physically plausible charge density *ρ*(**r**). Clearly, the irrotational current density vector **J**(**r**) differs from zero whenever the conductivity is different from zero; that is, it can extend outside the region *B* (i.e., “beyond the brain”). This may cast doubts on the physiological interpretability of the irrotational source model. In this paper we discuss this issue using some formal and/or intuitive arguments based on the Helmholtz decomposition of two source models: a dipole and a pure irrotational source.

In particular, we will demonstrate that, as suggested by (2), it is the divergence of **J** that must be confined to (i.e., different from zero only in) the brain region *B* and not **J** itself and that it is therefore the divergence of the current which constitutes the appropriate measure of the spatial extent of the generators, providing an interesting biophysical constraint, never used thus far, for the solution of the bioelectromagnetic inverse problem.

The paper is structured as follows: In Section 2 we briefly recall the Helmholtz decomposition (HD) as the main tool to analyze the current density vector (CDV) of the primary current and Dirac's delta function to represent “the current density vector of the dipole” (which, for simplicity we will call just “the dipole”). We will provide, for the first time, the formal derivation of the HD of the dipole and of a pure irrotational source. After noting that both, the irrotational and the solenoidal part, might extend all over the space even for concentrated sources like the dipole, we show that, for both sources, it is indeed the divergence of the irrotational part (which is the same as the divergence of the CDV) that remains confined to a region, confirming our initial guess based on (2). We show also that the dipole is solenoidal and irrotational everywhere in the 3D space except at its location and that there is no need for a pure solenoidal part to compensate for a pure irrotational part exiting the region where the primary current is expected to be confined to. After clarifying the physiological soundness of the irrotational source model (ISM) we will, in Section 3, show that the ISM remains valid far beyond the (quasi) static approximation as the only possible source of electrophysiological measurements in inhomogeneous, anisotropic, and dispersive media. To our knowledge, this is the first time these results are presented in the literature.

Unless otherwise stated, we will denote vectors and matrices by bold uppercase letters except the spatial variables that will be denoted by bold lowercase letters (e.g., **r** or **q**) or the material parameters that will be denoted by *ε*, *σ*, and *μ*. In standard equations where there is no place for confusion the dependence with the spatial variable will be omitted; that is, instead of ∇_**r**_ or *V*(**r**) or **A**(**r**) we will write just ∇, *V*, or **A**. For further details about the notation and conventions see the Appendices.

## 2. The Helmholtz Decomposition of the Dipole and the Irrotational Source

### 2.1. Helmholtz Decomposition Theorem

(Also called the fundamental theorem of vector calculus): let *B* ⊂ *ℜ* ^3^ be a regular domain in the sense that its boundary is the union of a finite number of smooth surfaces (i.e., *C* ^1^ surfaces). Then any vector field **J**(**r**) continuous in the closure of *B* and with continuous partial derivatives inside *B* can be uniquely expressed as the sum of an irrotational field and a solenoidal field.

Typically stated for isotropic media it has been also demonstrated for anisotropic media [4]. More relevant for this paper, it has been also extended [5] to infinite domains and/or to the case when the field **J**(**r**) has a finite number of isolated singular points and without any condition on the growth of **J**(**r**) when **r**→*∞*. Here we make use of the decomposition for the infinite and homogeneous medium sometimes called the free space, that is, *ℜ* ^3^.

The existence of HD is closely related to the solution of Poisson's equation (A.4). That is, the decomposition of the vector field **J**(**r**) will always be possible if there exists a field **C**(**r**) with continuous second order derivatives in *B* such that

(3) $$\begin{array}{c} J\left(\;r\;\right)=\nabla^{2}C\left(\;r\;\right). \end{array}$$

In effect using (A.2) we can rewrite **J**(**r**) as

(4) Jr∇∇·Cr−∇×∇×Cr=∇φr+∇×Ar,with  ∇·A≡0.

To achieve the decomposition we take alternatively the divergence and the rotor of (4) to obtain Poisson's equation for *φ* (using ∇·∇≡∇^2^) and **A** (using (A.2) and ∇·*A* ≡ 0), respectively, that, solved according to (A.4), yields

(5) φr−14π∫B∇p·Jpr−pdpAr=14π∫B∇p×Jpr−pdp.

This decomposition is unique up to an additive constant [6]. The uniqueness of this decomposition follows from the uniqueness of the solution to Poisson equation with Neumann boundary conditions.

In the electrodynamics literature it is usual to associate *φ* with the scalar potential and **A** with the vector potential. Consequently, Helmholtz decomposition lends itself to an interpretation of electrodynamics fields as related to spatial derivatives of potentials. However, a formal derivation of the expression for the solenoidal and irrotational components of different source models in terms of the delta formalism is missing from the literature. We provide next a formal derivation of the irrotational and solenoidal components of the fields linked to two sources of electrophysiological interest: the dipole and an extended pure irrotational CDV. This derivation serves to clarify several aspects linked to the spatial extent of a CDV, its solenoidal and irrotational components, and the link they have with the generators of the electromagnetic fields.

### 2.2. The Dipole

In lay terms a dipole at location **q** and dipolar moment **M** corresponds to a CDV that is zero everywhere except at point **q** where it takes the value of the dipolar moment. This is intrinsically associated with the informal, yet largely used, view of the Dirac's delta function located at point **q** as a function, in the classical sense, that is zero everywhere except at **q** where “the mass” is concentrated (for the formal definition see Appendix B); that is,

(6) $$\begin{array}{c} J_{d}\left(\;r\;\right)=M\delta \left(\;r,q\;\right). \end{array}$$

The informal interpretation of *δ* intuitively suggests (but does not prove) that the dipole (6) is irrotational and solenoidal everywhere where *δ* is zero (i.e., everywhere except at **q**). Yet, a more mathematically oriented reader might find difficulties in accepting results derived from the delta which is only defined in the sense of distributions. Consequently, we will use a more rigorous treatment of the *δ*, based on the definition and properties described in Appendix B to demonstrate this fact and derive the irrotational and solenoidal components of the dipole.

We remind the reader that in this paper the term “the dipole” designates “the current density vector of the dipole” as expressed by (6).

Since the dipole has a single singular point (at **q**) and behaves like the null function in every domain not containing the point **q** we can say that it satisfies all the necessary conditions for the computation for the HD.

### 2.3. Decomposition of the Dipole

In the following we assume that we have a region *B* (e.g., the brain), inside the infinite and homogeneous space *ℜ* ^3^, where the current density vector of the primary currents is confined to and therefore containing, in our case, the point **q** where the dipole is located. Note that, in that case, integrals over *ℜ* ^3^ are equivalent to integrals over *B*. Then we can compute the Helmholtz decomposition of the dipole in (6) using expressions (5). For the irrotational part we have (since **M** is independent of **p**)

(7) φr−14π∫B∇p·Mδp,qr−pdp=A.5−M4π·∫B∇pδp,qr−pdp=B.314πM·∇q1r−q

(8) φr14πM·r−qr−q3.

Taking the gradient of (8) yields the irrotational part of the dipole **I**_*d*_(**r**):

(9) $$\begin{array}{c} I_{d}\left(\;r\;\right)=\frac{1}{4\pi {\left|r-q\right|}^{3}}\left[\;M-\left(\;r-q\;\right)\frac{3M\cdot \left(r-q\right)}{{\left|r-q\right|}^{2}}\;\right]. \end{array}$$

Given that in electrostatics the electric field is the gradient of the electrostatic potential, that is, **E**(**r**) = −∇*V*(**r**), it is not surprising that *φ*(**r**) and −**I**_*d*_(**r**) are, respectively, the potential *V*(**r**) and the electric field **E**(**r**) generated at point **r** by a dipole at location **q**.

Equation (9) tells us that the irrotational component of a dipolar source has a well defined physical interpretation in terms of the electric field it produces. Obviously, both the electrostatic potential and the electric field extend all along the space and well beyond the point **q** where the dipole is located.

Note that the divergence of **I**_*d*_(**r**) is the Laplacian of potential *φ*(**r**) in (8), and direct calculation shows that this Laplacian is zero for **r** ≠ **q** and becomes indefinite at **r** = **q** where the dipole is placed. Thus, independently of the way we interpret what happens at **r** = **q** (e.g., the infinite Laplacian field predicted by the informal meaning of the *δ*), it is clear that the potential (8) is generated by what happens inside the region *R* = {**q**} ⊂ *B*. However, the divergence of the irrotational part of the dipole (9) is confined, as the dipole itself (6), to this very same region *R* = {**q**} where the primary current is known to be concentrated. This divergence, also known as the Current Source Density (CSD), coincides with the sources and sinks of the field and is traditionally estimated in neuroscience, under the quasi static approximation, by the Laplacian of the electrostatic potential. Thus, since the spatial extent of the dipolar generator is given by the divergence of its irrotational part we suggest that this is the quantity to be minimized in inverse solutions to retrieve focal sources.

In general the solenoidal part can be obtained by subtraction of the irrotational part (9) to the vector field we are decomposing, that is, (6) in our case. Here we compute it using the definition (5) and considering that **M** is independent of **p**; that is,

(10) Ar14π∫B∇p×Mδp,qr−pdp=A.5−M4π×∫B∇pδp,qr−pdp=B.314πM×∇q1r−q

(11) Ar14πM×r−qr−q3.

Taking the rotor of (11) we obtain the solenoidal part of the dipole **S**_*d*_(**r**) as

(12) $$\begin{array}{c} S_{d}\left(\;r\;\right)=\frac{1}{4\pi {\left|r-q\right|}^{3}}\left[\;\frac{3M\cdot \left(r-q\right)}{{\left|r-q\right|}^{2}}\left(\;r-q\;\right)-M\;\right]. \end{array}$$

Once again, as expected from magnetostatics, the vector potential (11) and its rotor (12) coincide with the magnetic field and the magnetic flux of a dipole (with unitary magnetic permeability).

We would like to remark that while the results of this section are, a posteriori (i.e., once we see them), not very surprising but expected for static fields, their mathematical proof using the Helmholtz decomposition of the dipole and/or the delta calculus is missing from the literature. The formal treatment presented here helps to clarify aspects of the source itself within the framework of the HD. All the formulas in this section were verified with the symbolic Matlab toolbox except for the integrals (7) and (10) that were computed analytically using formulae in Appendix B.

In short, results in this section show that from the decomposition of dipole as **J**_*d*_(**r**) = **M** *δ*(**r**, **q**) = **I**_*d*_(**r**) + **S**_*d*_(**r**) it follows that

(D1) theoretically, the only part of a dipole able to generate electric potentials (7)-(8) is **I**_*d*_(**r**); note that *φ*(**r**), defined by (7), is zero for divergenceless fields and the solenoidal component fulfills ∇·**S**_*d*_(**r**) ≡ 0 as the divergence of a rotor is always zero;
(D2) while the dipole denotes a source confined to location **q**, the irrotational component **I**_*d*_(**r**) and the solenoidal component **S**_*d*_(**r**) extend all over the region *B* and beyond it to all those parts of the space where the conductivity and permeability are different from zero (*μ* = *σ* = 1 in our case);
(D3) the spatial extent of a dipolar primary current is defined by the divergence of its irrotational part;
(D4) even if we computed the irrotational and the solenoidal parts separately using the defining equations (5) the results still satisfy that **I**_*d*_(**r**) + **S**_*d*_(**r**) ≡ 0 for all **r** ≠ **q**; that is, as expected from the intuitive definition of a dipole, the current density vector of a dipole **J**_*d*_(**r**) is really zero for all **r** ≠ **q**; as a consequence if **r** ≠ **q**, we have **I**_*d*_(**r**) = −**S**_*d*_(**r**) and then both parts are, up to a sign change, the gradient of the harmonic function *φ*(**r**).

In summary, the current dipole is electrically visible because it “contains” a distributed irrotational source **I**_*d*_(**r**) proportional to its electric field that, like the electric potential, extends all over the space. Nevertheless the divergence of **I**_*d*_(**r**) (i.e., exactly the same as the divergence of the dipole (6)) remains confined to the region *R* = {**q**}.

### 2.4. Decomposition of a Pure Irrotational Source

The analysis of the irrotational and solenoidal components of a distributed irrotational current might be also of some neurophysiological interest. Indeed, any current expressed as the gradient of another scalar field (e.g., ionic concentrations or purely ohmic currents) is necessarily irrotational. Therefore, in this section we compute the Helmholtz decomposition of the pure (because it contains no solenoidal part) irrotational source of (1). This function satisfies all necessary conditions of the HD theorem since it is continuous and has continuous derivatives all over the space. Then, since the rotor of a gradient is always zero the use of (5) yields

(13) φr−14π∫R3∇p·Jpr−pdp=2−14π∫Bρpr−pdp=1Vr,Ar0.

Thus, the irrotational component of a distributed irrotational current coincides with the original current density vector (1). As expected, the solenoidal part is zero everywhere; that is, **J**(**r**) = **I**_*i*_(**r**) + **S**_*i*_(**r**) with

(14) Iir∇Vr=Jr,Sir0.

From this decomposition it follows that

(I1) as for the dipole, a distributed irrotational source contributes to the EEG measurements via its irrotational part **I**_*i*_(**r**);
(I2) as for the dipole, the irrotational component **I**_*i*_(**r**) extends all over the space even if contrarily to the dipolar case the current density vector **J**(**r**) is not confined to any region;
(I3) however, according to (2), the divergence of the irrotational part ∇·**I**_*i*_(**r**) remains confined to the expected region *B* (i.e., the brain) containing the generators;
(I4) since the current density vector is not (a priori) zero anywhere, the irrotational components **I**_*i*_(**r**) and **S**_*i*_(**r**) are not canceling each other at any place; however, **I**_*i*_(**r**) changes qualitatively from the gradient of a nonharmonic function (*V*(**r**)) inside *B* to the gradient of a harmonic function outside *B* (the same *V*(**r**)!).

In summary, the irrotational part of a pure irrotational current source density vector is the same current density vector and extends all over the space. However, as for the dipole, the divergence of **I**_*i*_(**r**) remains confined to the region *B* (i.e., the brain) expected to contain the generators.

## 3. The Irrotational Source Model in Complex Media

The use of the quasi static approximation in the solution of the EEG inverse problem has been motivated by the range of frequencies of the EEG and the electrical properties of the media [7]. On this basis we might wonder if the ISM initially introduced for the (quasi) static approximation is also compatible with a more complete electrodynamics modeling of the EEG within more complex media. In the following we discuss the applicability of the ISM, considering both physically plausible models and mathematically treatable problems. Since we are mainly interested in the reconstruction of the sources from the EEG we will consider the cases where the electric and magnetic fields can be separated into independent equations. Separation into independent equations is not possible when both the magnetic permeability *μ* and the dielectric permittivity є are tensors. Fortunately, this situation “is not a case of primary importance” [8]. Then, following the tradition in neuroscience, we will consider the case where a magnetic permeability is a simple scalar independent of the space while the dielectric properties are expressed by tensors. Even if the models presented here are far away from the customary quasistatic EEG analysis we would note that this section is not to advocate a given mathematical model (like waves versus statics or any others) but just to show that the irrotational source model is not a simple consequence of, and therefore it is not limited to, the quasistatic approximation.

To start we consider an inhomogeneous (i.e., *ε* and *σ* depend on the location), anisotropic (i.e., *ε* and *σ* change with the spatial directions), and dispersive (i.e., *ε* and *σ* depend on frequency) lossy dielectric medium. In this case, *ε* and *σ* are represented by 3 × 3 square matrices (rank two tensors) and *μ* is a simple real number (scalar).

The conductivity and the permittivity tensors (or scalars) can be combined to obtain the complex permittivity as follows:

(15) εcr,w=εr,w−jσr,ww=εr,w+σr,wjwwith  j=−1.

On the other hand, it has been noticed [8] that Maxwell's equations are redundant in the sense that, from an axiomatic viewpoint, it is sufficient to consider the two rotor Maxwell equations together with the charge continuity equation to fully describe the behavior of the fields. Directly writing the two rotor Maxwell equations in the frequency domain to account for the presence of dispersion (frequency varying permittivity) we obtain

(16) ∇×Hr,wjwεr,w·Er,w+σr,w·Er,w+Jr,w=jwεcr,wEr,w+Jr,w

(17) ∇×Er,w−jwμHr,w,

where *ε*_*c*_ and *σ* are 3 × 3 matrices (rank two tensors) with elements that depend on frequency *w* and space **r**, *μ* is a scalar, and **J** denotes the unknown primary current density vector that produces the electric field and thus the EEG measurements. Note that the term *jw* appears in the right-hand term of (16) and (17) because differentiation in the time domain corresponds to multiplication by *jw* in the frequency domain. This term reflects the fact that, on this derivation, temporal variations of the fields are not anymore neglected; that is, the quasistatic approximation has been dropped.

Since **B** = *μ* **H** is a solenoidal field (i.e., ∇·**B** = 0) then there is a field **A** such that **B** = ∇×**A**. Thus, we can introduce the magnetic vector potential **A** to represent the fields **H** and **E** as

(18) Hr,w=1μ∇×Ar,wEr,w=1jwμεc−1r,w∇×∇×Ar,w−μJr,w,

where the expression for **E** is obtained taking the rotor of **H** in (18) and equating it to the rotor of **H** in (14).

Substituting **H** from (18) into (17) and equating it to the rotor of **E** (18) we conclude that **A** must fulfill

(19) ∇×εc−1r,w∇×∇×Ar,w−w2μAr,w−μεc−1Jr,w=0.

Since ∇×**Z** = 0 implies that there is a function *φ* and a constant *k* such that **Z** = *k*∇*φ*, we can then introduce the scalar function *φ* (with constant *k* = −*jwμ* conveniently selected for the matter of dimensions but independent of the spatial variable** r**) fulfilling

(20) ∇×∇×Ar,w−w2μεcr,wAr,w−μJr,w=−jwμεcr,w∇φr,w.

Taking the divergence on both sides of (20) and arranging terms leads to

(21) ∇·εcr,w∇φr,w+jw∇·εcr,wAr,w=∇·Jr,wjw.

Equation (21) already shows that the only part of the unknown current density vector **J** that contributes to the potential *φ* (i.e., to the EEG measurements) is the irrotational part of **J**. Indeed, from the Helmholtz decomposition of the primary current, **J**(**r**) = **I**(**r**) + **S**(**r**) with ∇·**S**(**r**) ≡ 0 follows that ∇·**J**(**r**) = ∇·[**I**(**r**) + **S**(**r**)] = ∇·**I**(**r**). Therefore, the right-hand term (the “source term”) in (21) will always be (irrespective of the shape of *ε*_*c*_) identical (up to the factor 1/*jw*) to the divergence of the irrotational component of **J**.

Equation (21) is not directly solvable for the arbitrary heterogeneous, anisotropic, and dispersive media considered thus far unless the fields are restricted to plane waves of a given polarization and the media are restricted to certain classes (e.g., birefringent) [8]. Yet, (21) can be simplified for simpler media such as an isotropic, homogeneous, and dispersive media where *ε* and *σ* reduce to single numbers depending only on the frequency and independent of the spatial direction or the spatial location. In this case the problem reduces to the nonhomogeneous Helmholtz equation in the frequency domain (equivalent to the wave equation on the time domain):

(22) $$\begin{array}{c} \nabla^{2}\varphi +\left(\;w^{2}\mu \varepsilon_{c}\;\right)\varphi =\frac{\nabla \cdot J}{jw\varepsilon_{c}}. \end{array}$$

To obtain (22) we have used the Lorenz gauge ∇·**A** = −*jwμε*_*c*_ *φ* where the wave number is defined as the first quadrant root of *κ* ^2^ = −*w* ^2^ *με*_*c*_ [8]. The necessity of fixing a gauge in electrodynamics arises from the nonunique definition of the scalar and vector potentials. Even if this discussion extends beyond the scope of this paper we consider it worth mentioning that the Lorenz Gauge (contrarily to the Coulomb gauge) naturally leads to wave equations for both the scalar and vector potentials. Consequently, within the Lorenz Gauge, not only the fields but also the potentials propagate at finite speeds in agreement with the causality principle.

In summary, from (21) or its simplified version (22), it is seen that the source term (right-hand term) that generates the scalar potential within complex materials, once the quasistatic approximation is dropped, is given by the divergence of the unknown **J**(**r**). Since the divergence of a solenoidal source is always zero, only irrotational currents can be source of the scalar potential.

## 4. Discussion

Section 2 presents the formal derivation of the irrotational and solenoidal parts obtained from HD for two different source models: a concentrated (dipolar) source and a distributed (purely irrotational) source. A posteriori, that is, after realizing the physical meaning of the irrotational and solenoidal components in terms of the electric field and magnetic flux, some of the derived conclusions might appear trivial. Yet, from the perspective of source modeling, they are not. Let us see why.

First, most of the apparently trivial interpretations of the results given here are based on treating the delta function (i.e., the dipole) as a function in the classical sense. This is formally incorrect since the delta function is not defined as a function of the space but only for integrals that include or not the location of the delta. In other words, an equality containing the delta function is to be interpreted only after integrating both terms according to the definition and properties of the delta function (e.g., Appendix B) resulting in an expression interpretable in the classical sense. This paper provides the irrotational and solenoidal parts of the dipole using later formalism.

Second, the adopted formalism seems to be the only way to clarify the apparent contradiction due to the difference between the spatial extent (i.e., the region where it is different from zero) of the primary current, the spatial extent of its irrotational part, and the spatial extent of the generators of the electric (i.e., EEG) data. In effect, independently of the spatial extension of an arbitrary primary current, its irrotational part extends as much as the potential it generates. However the solenoidal part can exist (e.g., the dipole) or not (e.g., the irrotational source) and extend all over the space (e.g., the dipole) while the generators (i.e., the divergence of the primary current) might be confined to a completely different region (e.g., the irrotational source). The apparent contradiction arises from using the wrong definition of the generators. Effectively, since the generator of the EEG is not the current density vector **J**(**r**) nor its irrotational part but its divergence, the divergence ∇·**J**(**r**) is the only element that correctly defines the spatial extension of the generators. Interestingly, this result holds true (see (21) and (22)) well beyond the quasistatic approximation and within extremely complex physical media as the source term is always given by the divergence ∇·**J**(**r**). Note that in such complex media and beyond quasistatic limits, computing the Laplacian of the potential is not sufficient to determine the generator extent (the divergence ∇·**J**(**r**)) since, in the more general case, the divergence is dependent on the vector potential (21) or on temporal derivatives of the potential (22). Consequently, this result is relevant not only for inverse modeling but for experimental and clinical neuroscience as well. For inverse modeling this result immediately provides an appropriate measure to optimize in order to arrive to more or less focal solutions. Within experimental neuroscience this result indicates that the association of the Laplacian estimated from multielectrode arrays to the sources and sinks (also called the CSD) of the field might be misleading, in particular, if the underlying geometry is complex and the quasistatic approximation is not valid. In clinical terms, this result might have important implications on the studies that try to define the extension of the epileptogenic area as needed for a supervised surgical resection.

From the decomposition of the dipole we observe that all over the space there is a solenoidal part that cancels out the irrotational part. However the decomposition of the pure irrotational source shows that this is not really a need but a particularity of the dipole. In general, for all electrically visible sources (i.e., that produces EEG), whenever the primary current density vector is zero, there will be a solenoidal part to cancel out the irrotational part. For the sake of the physical interpretation, in the analysis presented here we have excluded the dipole location **q**. However, we should mention that there are straightforward ways to compute “the value” of the irrotational part and the solenoidal part at this point. Unfortunately these values lack a physical interpretation since they are just fractions (i.e., 1/3 and 2/3) of the dipole CDV (6). In simple terms this corresponds to decomposing the delta as a combination of fractions of the delta.

Section 3 shows that the irrotational source model (ISM) is also valid for homogeneous isotropic dispersive media. In fact, since the assumption of homogeneity was only used after (21), it results from (21) that the ISM is also compatible with inhomogeneous anisotropic dispersive media, which corresponds to one of the more complex media we can figure out. Indeed, (21) and (22) show that the only part of the primary current that contributes to the scalar potential, in complex materials and beyond quasistatic limit, is given by the divergence of the primary current. Since the solenoidal component of a current is by definition divergenceless it cannot behave as a source of the scalar potential (e.g., the EEG) no matter how complex the media is. Therefore, Section 3 shows that the ISM is completely independent of the quasistatic assumptions and is indeed valid within a more general electrodynamics formulation of the EEG inverse problem where neural tissue is modeled with its full complexity (i.e., as an inhomogeneous anisotropic and dispersive media).

## 5. Conclusion

In this paper we have discussed the sources of the EEG making emphasis on the irrotational source model (ISM) originally proposed in [1] as a physically plausible model for EEG sources. We have used the Helmholtz decomposition to show that the EEG can only be generated by irrotational sources. In particular we provide, for the first time, the decomposition of the dipole (6) and a pure irrotational source (1) into an irrotational and a solenoidal part to conclude that while the irrotational part extends all over the space (i.e., outside the region expected to contain the primary current), the solenoidal part may (or not) have the same extension. Outside that region and within the quasistatic limits, the irrotational part is the gradient of a harmonic function. Importantly, even if both parts extend (or not) all over the space, only the divergence of the irrotational part, corresponding to the generators of the EEG, remains always confined to the expected region of the primary currents. Thus, it is the only element that can be used to reliably estimate the extension of the generators. Consequently, the irrotational source model is physiologically meaningful as long as its divergence remains confined to the brain volume.

We have also shown that the ISM is compatible with more general electrodynamics models of the EEG including inhomogeneous anisotropic dispersive media and thus that it is not limited to the quasistatic approximation.

In summary, from a rigorous point of view, the irrotational source model of ELECTRA corresponds to the estimation of the irrotational part of the unknown primary current** J** from scalp EEG data. This does not mean that sources inside the brain are irrotational. It simply means that EEG measurements are insensitive to the solenoidal part of the primary current. This might look as a limitation but it is not. Every nonsilent current contains an irrotational part that makes it electrically visible. Thus, it seems that pure vortex sources are more likely to exist in the mathematical universe than in a real brain. In conclusion, the irrotational source model not only is an efficient way to simplify the solution of Maxwell equations, when only electrical data is available, by reducing the complexity of the inverse problem from the estimation of a vector field to the estimation of a scalar field, but also is compatible with the different complex media (e.g., inhomogeneous anisotropic), with a more complete electrodynamics approach (e.g., wave or Helmholtz equations) and with the diverse electrophysiological recording scales.

## Appendices

Notations examples and definitions for the mathematical elements used in the paper and the appendices are the following:

- Points in 3D Euclidian space *ℜ* ^3^ are **r** ≡ {*r*_*x*_, *r*_*y*_, *r*_*z*_}^*t*^.
- Vector fields in *ℜ* ^3^ are **A**(**r**) ≡ {*a*_*x*_(**r**), *a*_*y*_(**r**), *a*_*z*_(**r**)}^*t*^.
- Scalar fields in *ℜ* ^3^ are *φ*(**r**).
- Nabla (or Del) symbol is ∇≡{∂/∂*x*, ∂/∂*y*, ∂/∂*z*} such that ∇·**A**, ∇×**A**, and ∇*φ* denote the divergence, the rotor (or curl), and the gradient operators acting on vector or scalar functions, respectively. The “·” and “×” denote the scalar (dot) and vector (cross) product of vectors and ∇^2^ **A** or ∇^2^ *φ* denote the Laplacian of a vector or scalar field.
- A vector field **A** with ∇·**A** = 0 is called* solenoidal* or divergence-free. A vector field with ∇×**A** = 0 is called* irrotational* or curl-free.

### A. Some Vector Identities Used in This Paper

Given vectors **A**, **B**, and **C**, then

(A.1) $$\begin{array}{c} A\times B\times C=B\left(\;A\cdot C\;\right)-\left(\;A\cdot B\;\right)C. \end{array}$$

Taking **A** = ∇ and **B** = ∇ and **C** as a 3D vector field we have a symbolic derivation (more mnemonic than maths) of the Laplacian of **C**:

(A.2) $$\begin{array}{c} \nabla \times \nabla \times C=\nabla \left(\;\nabla \cdot C\;\right)-\nabla^{2}C. \end{array}$$

In particular for the Laplacian of the Green function of the infinite medium we have

(A.3) $$\begin{array}{c} \nabla_{r}^{2}\cdot \left(\;\frac{1}{\left|r-p\right|}\;\right)=-4\pi \delta \left(\;r,p\;\right). \end{array}$$

The solution of Poisson's equation for the free space, that is, the infinite medium without boundaries, **J**(**r**) = ∇^2^ **C**(**r**), is (idem for scalar fields)

(A.4) $$\begin{array}{c} C\left(\;r\;\right)=-\frac{1}{4\pi }\overset{}{\underset{R^{3}}{\int }}\frac{J\left(p\right)}{\left|r-p\right|}dp. \end{array}$$

If **M** is a constant vector, then

(A.5) ∇·MφrM·∇φr∇×Mφr−∇×φrM=−M×∇φr.

### B. The Delta Function

Let *φ*(**r**) be a scalar function with compact support (i.e., different from zero in a closed and bounded set of points *B*) and the primed *φ*′(**r**), the result of first order differentiation operations (e.g., gradient). Note that since *δ* behaves as a compactly supported function, similar statements are valid for not compactly supported functions *φ*, as suggested by (B.1), where *φ* ≡ 1 extends all over the space. Then, we denote by *δ*(**r**, **q**) or *δ*(**r** − **q**) the generalized function (sometimes also called functional, distribution, or measure) fulfilling the following equations for any region *B* ⊂ *ℜ* ^3^ containing the point** q**:

(B.1) ∫R3δr,qdr∫Bδr,qdr=1

(B.2) ∫R3ϕrδr,qdr∫Bϕrδr,qdr=∫Bϕrδq,rdr=ϕq

(B.3) ∫R3ϕrδ′r,qdr∫Bϕrδ′r,qdr=−∫Bϕr′δr,qdr=−ϕ′q.
